# Quantifying crustal thickness over time in magmatic arcs

**DOI:** 10.1038/srep17786

**Published:** 2015-12-03

**Authors:** Lucia Profeta, Mihai N. Ducea, James B. Chapman, Scott R. Paterson, Susana Marisol Henriquez Gonzales, Moritz Kirsch, Lucian Petrescu, Peter G. DeCelles

**Affiliations:** 1 University of Arizona, Department of Geosciences, AZ 85721, USA; 2 University of Bucharest, Faculty of Geology and Geophysics, Bucharest, 010041, Romania; 3 University of Southern California, Department of Earth Sciences, Los Angeles, CA 90089, USA

## Abstract

We present global and regional correlations between whole-rock values of Sr/Y and La/Yb and crustal thickness for intermediate rocks from modern subduction-related magmatic arcs formed around the Pacific. These correlations bolster earlier ideas that various geochemical parameters can be used to track changes of crustal thickness through time in ancient subduction systems. Inferred crustal thicknesses using our proposed empirical fits are consistent with independent geologic constraints for the Cenozoic evolution of the central Andes, as well as various Mesozoic magmatic arc segments currently exposed in the Coast Mountains, British Columbia, and the Sierra Nevada and Mojave-Transverse Range regions of California. We propose that these geochemical parameters can be used, when averaged over the typical lifetimes and spatial footprints of composite volcanoes and their intrusive equivalents to infer crustal thickness changes over time in ancient orogens.

Magmatism at subduction zones originates in the asthenosphere above the downgoing slab and is a result of partial melting of the mantle in the presence of aqueous fluids released from the slab^1^. These processes produce primary basaltic melts, which then ascend into the overlying plate. Global geochemical trends of mafic rocks found in arcs are influenced in part by heterogeneities in these primary magmas^2^ and are complemented by subsequent fractionation of these liquids in the crust^3^, as well as several assimilation mechanisms of the pre-existing upper plate crust^4^. The end result of arc magmatism is a crustal column dominated by magmatic products^5^,^6^ and an average upper crustal composition that is variable in composition but on average is andesitic, regardless of whether the subduction is intra-oceanic or if the upper plate is continental. Island arcs tend to be more mafic than continental arcs in most places^7^. Certain major element oxides of mafic arc rocks (e.g. NaO, CaO) correlate with crustal thickness at global scales^8^,^9^ as do some trace elemental ratios^10^,^11^ (La/Yb or Ce/Y), likely indicating their average depth of melting in the mantle and thus indirectly placing limits on crustal thickness. Intermediate rock geochemical parameters involving major elements or their ratios, such as silica or K_2_O/Na_2_O may also correlate with crustal thickness at global scales^8^,^12^,^13^, however most such correlations break down where crustal thicknesses exceed 45 km. Perhaps the only major elemental oxide that correlates with crustal thickness at global scales is MnO, but the magnitude of this oxide’s variation in calk-alkaline rocks is small and the correlation is weak. In addition, there are hypothesized trace elemental parameters in intermediate rocks that could be sensitive to crustal thickness in arcs at regional scales (e.g. the slope of REEs^14^, or Sr/Y^15^).

Chapman *et al*.^16^ showed that tonalite (andesite) and granodiorite (dacite) whole-rock Sr/Y varies regionally with crustal thickness in the western North American Cordilleran interior. As an extension of that work, here we show that two trace elemental ratios, Sr/Y and La/Yb can be used as crustal thickness proxies in low magnesium intermediate calc-alkaline rocks (55–68 wt% SiO_2_) covering the compositional range of andesites and dacites and their intrusive equivalents (hereafter referred to as *intermediate* rocks), when larger global and regional datasets are averaged out. We accomplish this first by using an existing large global database for geochemistry of geologically young, Pliocene to modern arc rocks and knowledge of global crustal thickness, followed by comparison of the global to a regional dataset, the southern Andean zone, where crustal thickness varies significantly along the strike of the volcanic arc front^17^. We then test our global and regional empirical correlations in a few ancient subduction margins where independent geologic constraints exist on variations in crustal thickness over time.

## Background

**Figure 1:**
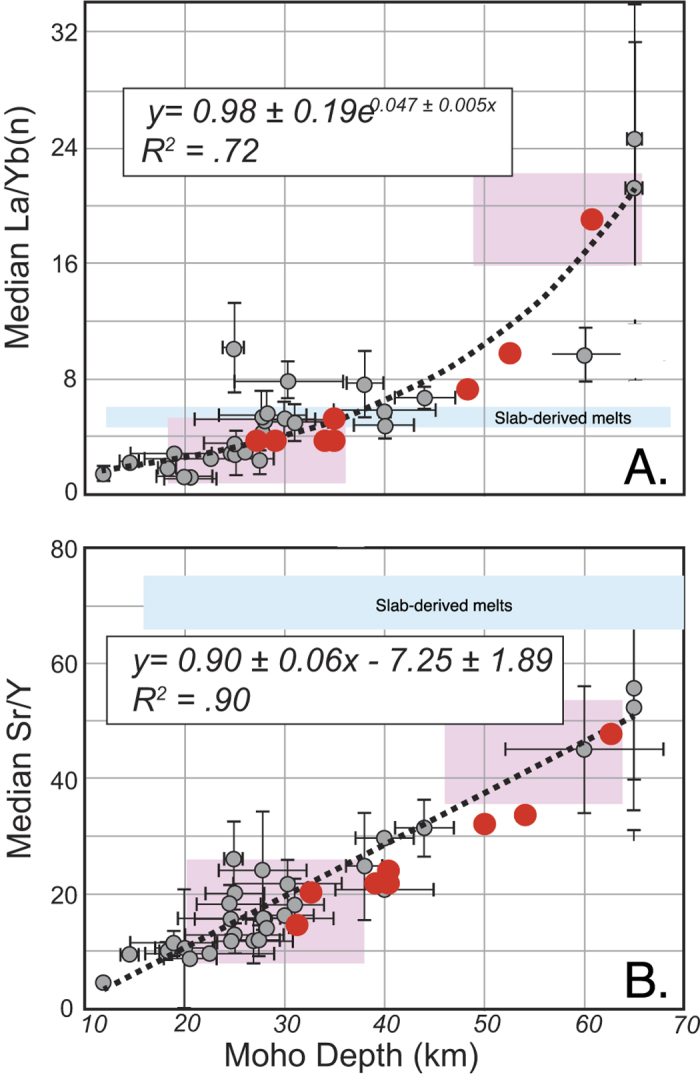
Whole-rock (A) La/Yb and (B) Sr/Y correlations with crustal thickness for Quaternary and modern subduction-related volcanic arcs. La/Yb ratios are normalized to chondritic values. Gray circles represent individual arc segments used for the global correlations (dotted line and empirical fits with R^2^ shown on each diagram). Data are from GEOROC and various geophysical estimates of crustal thickness (See Methods). Red dots are average values for individual volcanoes of the northern part of the Southern Volcanic Zone of the Andes, where crustal thickness varies regionally by almost a factor of two; standard deviations are represented by the size of the individual data points. The range of slab-derived melts La/Yb and Sr/Y is shown in light blue assumes that the mafic oceanic crust id the only part of the slab that melts and is calculated based on typical MORB trace elemental concentrations and partition coefficients for these elements involving intermediate melt and an eclogitic residue. Pink boxes represent a predicted range of La/Yb and Sr/Y for thin versus thick sub arc crust, using partition coefficients from reference (20) and average residual sub arc assemblages for thin (gabbronorite residues from the Ordovician Famatinian arc, Argentina^23^) and thick (pyroxenites with and without garnet from the Sierra Nevada arc, California^22^) arcs respectively. La/Yb throughout this paper are normalized to chondritic values^29^.

Sr/Y and La/Yb are commonly used in petrology to qualitatively infer depths of magmatic diversification. They can distinguish deep from shallow fractionation processes due to differences in the partition coefficient between these elements in various residual phases and intermediate melt^11^,^14^,^18^. During partial melting of lower crustal igneous rocks such as gabbros and their metamorphic equivalents or during magmatic fractionation of mantle-derived mafic magmas, Sr is preferentially incorporated into the residue at low pressures (<~1.0 GPa) where it strongly partitions into plagioclase. However, at higher pressures (>1.2 GPa), where plagioclase is less abundant and with increasing pressure eventually becomes unstable, Sr preferentially enters the liquid phase. On the other hand, Y is incompatible at low pressures, but readily partitions into garnet at high-pressure. The presence of amphibole, a mineral that can both be consumed and generated during the formation of a residual lower part of a batholith^19^,^20^,^21^ will also elevate the Sr/Y ratios (Fig. 1), although separating amphibole from garnet effects using these elements alone has proven to be difficult. As a result, Sr/Y may act as a pseudo-barometer reflecting the average depth at which magmatic diversification occurred. A larger Sr/Y ratio in the volcanic and batholithic parts of an arc signifies a greater pressure or depth. Predicted values for this ratio are shown in Fig. 1, based on typical concentrations in residual sub-arc rocks^22^,^23^ and known residue-intermediate melt partition coefficients from the GERM (Geochemical Earth Reference Model) online database of partition coefficients between various minerals in equilibrium with liquids (http://earthref.org/KDD/). We use averages of partition coefficients for dacitic and andesitic liquids referenced on that online database.

A similar argument can be made for the ratios of light (L) to heavy (H) rare earth elements (REE); we use the La/Yb ratio in this analysis. Elevated La/Yb ratios are predicted in rocks that are fractionated out of garnet and/or amphibole rich residues in thick arcs, whereas La/Yb ratios are lower in thin arc residues^14^ (Fig. 1). Observations in exposed tilted sections of magmatic arcs have shown that the bulk of compositional diversity is achieved in the deeper parts of arc crust, where thermal efficiency is high^4^,^17^,^24^, regardless of crustal thickness. Therefore geochemical parameters can indirectly map out various fractionating phases, although clearly one expects complexities that need to be averaged out over time- and length-scales larger than an individual eruption.

Melting of subducted oceanic crust can also generate intermediate magmas^25^, and the principal reservoir that can be responsible for partial melting is the oceanic crust, which is the sum of various mafic igneous rocks, totaling about 6 km in thickness in most oceanic areas. The La/Yb of these melts will be low reflecting the light rare earth element depleted nature of mid-ocean ridge basalts (Fig. 1). Sr/Y, on the other hand should be >60 in the case of intermediate rocks generated by slab melting compared to Sr/Y of 30–50 for intermediate rocks extracted from arc roots from thick crust because of the higher Sr in altered mid-ocean ridge basalt compared to basalts/gabbros from sub arc environments^23^,^26^ (Fig. 1). The positive correlation between Sr/Y and La/Yb in virtually all intermediate arcs argues strongly for the fact that the majority of modern Pacific arc magmatic products owe their compositional diversity of the trace elements investigated here to partial melting or crystal fractionation in the crust^27^,^28^ and that slab melting plays at most a secondary role.

## Results

A large number of previously published whole rock geochemical analyses were used for this study; the methods section at the end of the manuscript details the selection of data and their manipulation.

### Global and regional correlations

We observe that for Pliocene to modern subduction-related intermediate rock compositions there is a positive correlation of global arc-averaged Sr/Y and La/Yb with crustal thickness (Fig. 1). The empirical fits to the global data are:


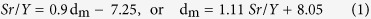



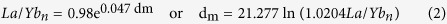


where *Sr/Y* and *La/Yb*_*n*_ are whole rock average ratios (and the subscript “n” for La/Yb implies that that ratio was normalized to chondritic values of McDonough and Sun^29^), and d_m_= crustal thickness or depth to Moho.

Our Sr/Y results are similar to the global Sr/Y correlation with crustal thickness of Chiaradia^15^; in that paper rocks spanning a wide range of compositions (from mafic to the most felsic) were mixed in the correlation. A limiting factor in this type of analysis is that sub-arc Moho depths obtained from seismic data may be underestimated because the crust-mantle boundary could be a complex transition involving mantle-like cumulates (e.g. gabbronorites transitioning to pyroxenites, with or without garnet^5^,^6^,^22^), rather than a change from typical lower crustal rocks to a peridotitic assemblage. Therefore, the sub arc crust may be thicker petrologically than seismically, an uncertainty less likely to occur in other tectonic environments.

We also show in Fig. 1 that similar trends can be seen at regional scale in the northern part of the Southern Andean volcanic zone, which is the only modern arc where significant changes in crustal thickness (from over 60 km to less than 30 km^17^) occur over a relatively short distance along strike. Regional data correlations show that when data from individual composite volcanoes data are averaged over the lifetime of the volcano (about 5 million years), the trace elemental ratios discussed here mimic global trends. Of the two ratios, La/Yb has less scatter in global and regional correlations, and is a better indicator of thicker crust in continental arcs. How these correlations using volcanic data apply to the subvolcanic and plutonic equivalents at depth is not straightforward. Limited comparisons of volcanic versus plutonic Sr/Y and La/Yb ratios in ancient arcs^7^ suggest that batholiths have Sr/Y ratios that may be somewhat higher (by ~10-15%) than the volcanic cover, whereas no systematic difference between volcanoes and batholiths is noticed in La/Yb.

### Applications to ancient orogens

**Figure 2:**
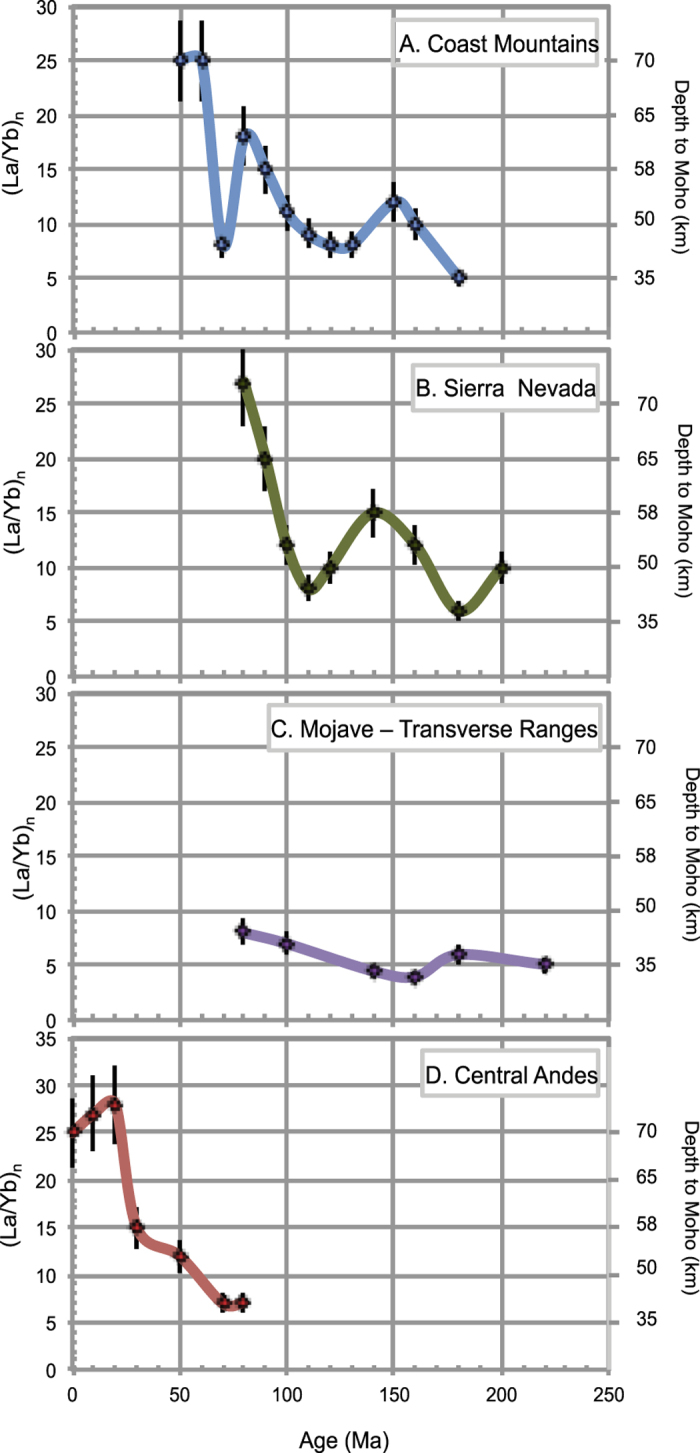
Average chondrite-normalized La/Yb versus time (million years) for several ancient subduction-related Andean, magmatic arcs. (**A**) Coast Mountains Batholith (52–55°N), (**B**) central Sierra Nevada batholith, California (35–38°N), (**C**) the Mojave-Transverse range segment of the Mesozoic California arc and (**D**) Central Andean frontal arc (15–24°S). Individual data points are 5 million year averages and median values for trace ratios with estimated 1-sigma error bars. See methods for data sources.

**Figure 3:**
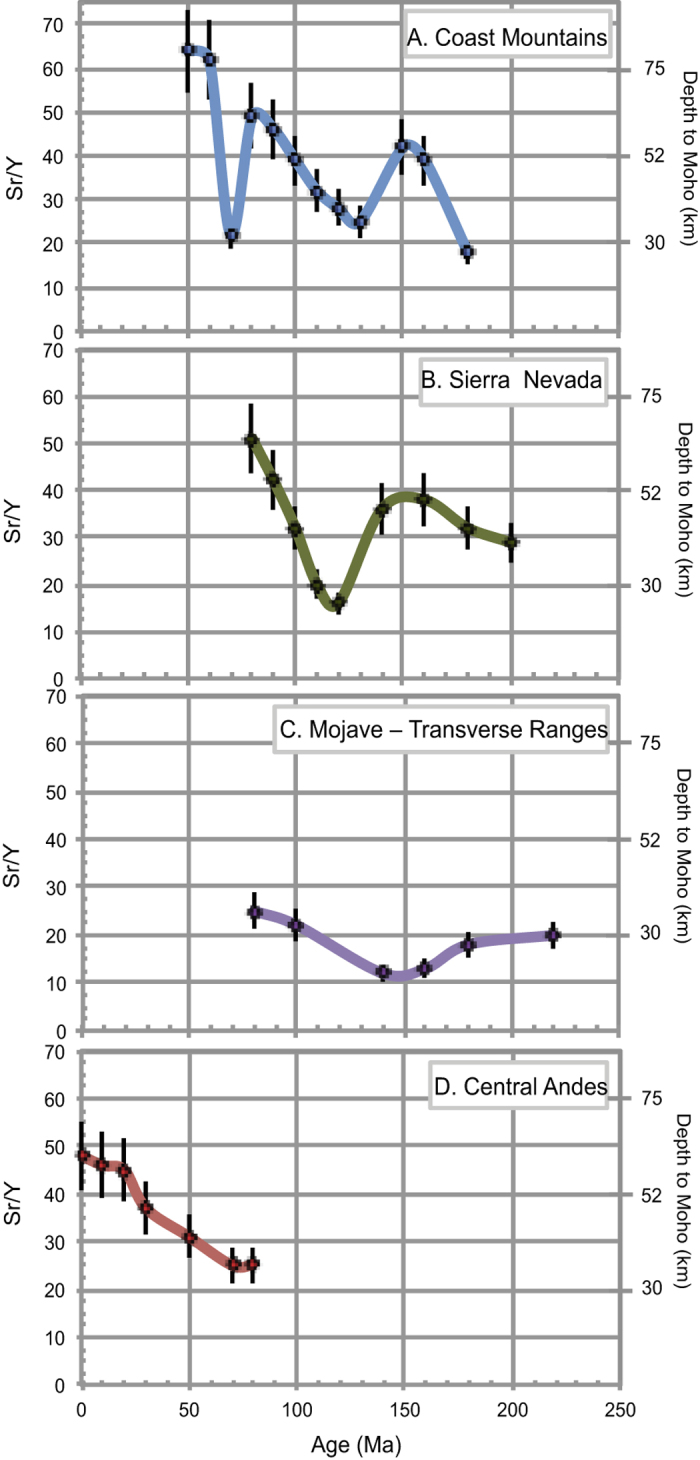
Average Sr/Y versus time (million years) for several ancient subduction-related Andean, magmatic arcs; various arc segments are as in Fig. 2.

Figures 2 and 3 show La/Yb and Sr/Y respectively as a function of time for several ancient magmatic arcs whose regional tectonic evolution is independently constrained by various geologic data. We average out rocks representing individual magmatic systems (composite volcanoes or their roots representing co-magmatic suites in a batholith). This typically corresponds to a 30 km spatial average and ~5 million years temporal average, although the numbers differ from region to region, depending on local particularities of magmatism and data availability. The two central Andes transects are consistent with the idea that the most recent crustal thickening began during an early Cenozoic orogeny (45–55 Ma), a process that continues through modern time^30^,^31^. The Coast Mountains batholith displays three significant pulses of Sr/Y and La/Yb increase, one during the Latest Jurassic, another during the Late Cretaceous and the last one during the Paleocene-Eocene, separated by periods of lower Sr/Y and La/Y. All three episodes of increase in these ratios correspond temporally to periods of crustal thickening in the arc’s foreland area^32^. The Mesozoic Sierra Nevada batholith of California shows a gradual increase in crustal thickness from the mid-Cretaceous on, also consistent with independent geologic constraints such significant crustal shortening in the retroarc thrust belt^33^. In both cases, the leading hypothesis for intra arc crustal thinning is thought to be due to episodes of lower crustal delamination occurring after significant crustal thickening, a sequence that is thought to occur cyclically in long-lived arcs^34^. In contrast to these arcs, the along strike segment of arc that formed south of the Sierra Nevada, represented by the Mojave-Transverse Ranges corridor, does not appear to have attained thick crust during the late Cretaceous, as determined from independent regional geologic observations^35^; our compiled data confirm that assertion.

## Discussion

Modern global and regional data and ancient regional examples lend confidence to the use of Sr/Y and La/Yb correlations in determining paleo-crustal evolution in magmatically active subduction orogens, such as the Cordilleran/Andean ones investigated here. In contrast, short lived subduction events in Mediterranean type environments and collision-related magmatism are far less understood petrologically; our data may apply to those tectonic environments but that has not been tested yet. As for *sui generis* subduction magmatism, such correlations averaging out several processes as well as length and timescales will inherently be subjected to large errors; they nonetheless seem to provide reasonable quantitative constraints on crustal thickness evolution in subduction-related orogens. We propose that episodes of thin versus thick crust can be distinguished in the geologic record of subduction-related arc magmatism using the geochemical ratios presented here.

## Methods

### Databases for modern arcs

We use whole rock analyses from 25 Plio-Quaternary Circum-Pacific volcanic arcs from the GEOROC database (http://georoc.mpch-mainz.gwdg.de/georoc/). This data set consisting of 18,200 data points was filtered to include rocks with SiO_2_ content (55–68 wt %) and MgO content (<4 wt %); the resulting number of individual points after this filtering was decreased to 9,800. This range of silica concentration was used to eliminate mafic rocks generated in the mantle, as well as high silica granites; in both cases, the distribution of the trace elements investigated here may reflect processes other than those presented in this manuscript and therefore may not be indicative of crustal thickness. We removed La/Yb and Sr/Y outliers from these data subsets using the modified Thompson Tau statistical method and calculated median values. Data with average Rb/Sr > 0.2 or Rb/Sr < 0.05 were discarded, providing a trace elemental filter for mantle-derived rocks or rocks formed by melting of pre-existing metasedimentary framework rocks, which could have been missed by the silica and magnesium filters (protocol as in Chapman *et al*.^16^). A total of 4,800 data points were used in the global correlations (Supplementary files 1 and 2). Crustal thickness estimates for the volcanic arcs used in this paper are also given Supplementary files 1 and 2. A regional database for the northern part of the South Andean Volcanic zone was also generated using GEOROC and filtered identically as for the global set. Averages for individual composite volcanoes spanning as much as 5 million years are used in the correlation in Fig. 1. Crustal thickness was calculated using the average elevations of the areas occupied by strato-volcanoes, assuming isostatic equilibrium at a horizontal scale of 50 km and an average crustal density of 2700 kg/m^3^.

### Data from ancient arcs

Only data with tightly constrained ages (<5 My error), most of which are determined by isotopic techniques were used here. Geochemical data on ancient arcs are from the central Andean data base^30^ (http://andes.gzg.geo.uni-goettingen.de). Data for the Coast Mountains, Sierra Nevada and Transverse Range-Mojave arc segments data are from NAVDAT, the North American Volcanic (and Intrusive) Data Base, or (http://www.navdat.org) supplemented by various published and unpublished data compiled by Scott Paterson and students. Filtering applied to these data is identical to that described above for the modern global and regional databases and Chapman *et al*.^16^. Filtered data were pooled into 5 million years time intervals and 50 km diameter spatial footprints in order to mimic typical average lifetimes and spatial distribution of modern volcanoes in subduction zones^7^.

## Additional Information

**How to cite this article**: Profeta, L. *et al.* Quantifying crustal thickness over time in magmatic arcs. *Sci. Rep.* **5**, 17786; doi: 10.1038/srep17786 (2015).

## Supplementary Material

Supplementary Information
