# Supplementary material for: Quantifying crustal thickness over time in magmatic arcs
Source: Sci Rep. 2015 Dec 3;5:17786. doi: 10.1038/srep17786 (PMC4668569; doi:10.1038/srep17786)
Supplement: Supplementary Information [file srep17786-s1.pdf]

Title: Quantifying crustal thickness over time in magmatic arcs
Authors: Profeta L., Ducea, M.N., Chapman, J.B., Paterson, S.R., Henriquez Gonzales S.M., Kirsch, M., Petrescu L., DeCelles P.G.

Supplementary Material 1: La/Y data

Arc	La/Yb median	stdev	DM km	stdev	DM Source
Aleutian	2.8	0.17	18.9	4.4	Zellmer, 2008
C. America	5.96	2.13	28	7	Zellmer, 2008
Cascades	7.7	2.37	38	1.9	Zellmer, 2008
CVZ	22.57	9.51	65	0.7	Zellmer, 2008
Guatemala	6.49	0.74	44	3	Lucke 2014
Honshu	3.7	0.48	30.2	3.6	CRUST 1.0
Izu-Bonin	1.43	0.45	20.5	2.7	Zellmer, 2008
Kamchatka	3.71	0.33	34.6	4.8	CRUST 1.0
Kurile	1.95	0.34	18.3	0.9	Zellmer, 2008
L. Antilles	3.13	0.49	24.7	0.7	Zellmer, 2008
Luzon	5.55	1.69	30.8	1.4	CRUST 1.0
Mariana	2.18	0.05	14.5	1	Zellmer, 2008
Mexican	7.85	1.3	37.2	9	CRUST 1.0
NE Aleutian	4.73	0.14	40	3	Eberhart-Phillips, et al., 2006
New Britain	2.54	0.54	27.1	4.3	CRUST 1.0
New Hebrides	3.61	0.85	25	3	Dimalanta et al., 2002
NVZ	9.54	2.95	54.4	4.7	CRUST 1.0
Ryukyu	2.78	0.39	24.5	3.4	Zellmer, 2008
S. Sandwich	1.32	0.51	11.8	0.1	Zellmer, 2008
S. Shetland	4.93	1.23	26.2	1.2	CRUST 1.0
Sulawesi	2.35	1.1	27.4	2.2	Zellmer, 2008
Sunda	4.42	1.03	27.8	1.8	Zellmer, 2008
SVZ	6.48	2	40	5	Yuan et al., 2006
Tonga	1.21	0.16	20	3	Zellmer, 2008

Suplementary table 1: La/Yb for individual arcs. DM – depth to Moho or crustal thickness. NVZ, CZ and SVZ are northern, central and southern volcanic zones of the Andes, respectively.

CRUSTAL THICKNESS
References for Moho data:

Dimalanta, C., Taira, A., Yumul Jr., G.P., Tokuyama, H., and Mochizuki, K., 2002, New rates of western Pacific island arc magmatism from seismic and gravity data: Earth and Planetary Science Letters, v. 202, p. 105-115.
Eberhart-Phillips, D., Christensen, D.H., Brocher, T.M., Hansen, R., Ruppert, N.A., Haeussler, P.J., and Abers, G.A., 2006, Imaging the transition from Aleutian subduction to Yakutat collision in central Alaska, with local earthquakes and active source data: Journal of Geophysical Research, v. 111, B11303, doi:10.1029/2005JB004240.

Lucke, O.H., 2014, Moho structure of Central America based on three-dimensional lithospheric density modelling of satellite-derived gravity data: International Journal of Earth Science, v. 103, p. 1733-1745, DOI 10.1007/s00531-012-0787-y.

Yuan, X., Asch, G., Bataille, K., Bock, G., Bohm, M., Echtler, H., Kind, R., Oncken, O., and Wölbern, I., 2006, Deep seismic images of the Southern Andes, in Kay, S.M., and Ramos, V.A., eds., Evolution of an Andean margin: A tectonic and magmatic view from the Andes to the Neuquén Basin (35°–39°S lat): Geological Society of America Special Paper 407, p. 61–72, doi: 10.1130/2006.2407(03).

Zellmer, G.F., 2008, Some first-order observations on magma transfer from mantle wedge to upper crust at volcanic arcs: Geological Society of London Special Publication 304, p. 15–31.

Model Crust 1.0 is from Laske et al database available at the following site accessed August 2015 http://igppweb.ucsd.edu/~gabi/crust1.html.

Laske, G., Masters., G., Ma, Z. and Pasyanos, M., Update on CRUST1.0 - A 1-degree Global Model of Earth's Crust, Geophys. Res. Abstracts, 15, Abstract EGU2013-2658, 2013.

GEOCHEMISTRY

Geochemical Data for Pliocene to Quaternary rock analyses used to calculate La/Yb. These analyses have been filtered to remove SiO2 wt. % < 55, SiO2 wt. % > 68, MgO wt. % < 1, MgO wt. % > 6, Rb/Sr > 0.2, Rb/Sr <0.05, and Sr/Y outliers using the Thomson Tau method. Data is from Geochemistry of Rocks of the Oceans and Continents database (GEOROC; http://georoc.mpch-mainz.gwdg.de/georoc/, accessed July 2015).

Title: Quantifying crustal thickness over time in magmatic arcs
Authors: Profeta L., Ducea, M.N., Chapman, J.B., Paterson, S.R., Henriquez Gonzales S.M., Kirsch, M., Petrescu L., DeCelles P.G.

Supplementary Material 2: Sr/Y data

Arc	Sr/Y median	stdev	DM km	stdev	DM Source
Aleutian	11.4	2.3	18.9	4.4	Zellmer, 2008
C. America	15.6	2.6	28.0	7.0	Zellmer, 2008
Cascades	24.8	9.4	38.0	1.9	Zellmer, 2008
CVZ	56.5	18.5	65.0	0.7	Zellmer, 2008
Guatemala	31.4	5.1	44.0	3.0	Lucke 2014
Honshu	11.7	4	30.2	3.6	CRUST 1.0
Izu-Bonin	8.7	1.3	20.5	2.7	Zellmer, 2008
Kamchatka	15.5	4.7	34.6	4.8	CRUST 1.0
Kurile	9.9	1.6	18.3	0.9	Zellmer, 2008
L. Antilles	11.8	1.4	24.7	0.7	Zellmer, 2008
Luzon	24	10.3	30.8	1.4	CRUST 1.0
Marianas	9.4	0.3	14.5	1.0	Zellmer, 2008
Mexican	21.6	4.5	37.2	9.0	CRUST 1.0
NE Aluetian	29.6	0.6	40.0	3.0	Eberhart-Phillips, et al., 2006
New Britain	9.5	1	27.1	4.3	CRUST 1.0
New Hebrides	20.1	5.6	25.0	3.0	Dimalanta et al., 2002
NVZ	43.4	11.2	54.4	4.7	CRUST 1.0
Ryukyu	18.2	3.5	24.5	3.4	Zellmer, 2008
S. Sandwich	4.5	0.3	11.8	0.1	Zellmer, 2008
S. Shetland	17.9	4.8	26.2	1.2	CRUST 1.0
Sulawesi	11.9	2.8	27.4	2.2	Zellmer, 2008
Sunda	15.7	2.9	27.8	1.8	Zellmer, 2008
SVZ	20.7	7.7	40.0	5.0	Yuan et al., 2006
Tonga	10.5	10.4	20.0	3.0	Zellmer, 2008

Suplementary table 1: Sr/Y for individual arcs. DM – depth to Moho or crustal thickness. NVZ, CZ and SVZ are northern, central and southern volcanic zones of the Andes, respectively.

CRUSTAL THICKNESS
References for Moho data:

Dimalanta, C., Taira, A., Yumul Jr., G.P., Tokuyama, H., and Mochizuki, K., 2002, New rates of western Pacific island arc magmatism from seismic and gravity data: Earth and Planetary Science Letters, v. 202, p. 105-115.

Eberhart-Phillips, D., Christensen, D.H., Brocher, T.M., Hansen, R., Ruppert, N.A., Haeussler, P.J., and Abers, G.A., 2006, Imaging the transition from Aleutian subduction to Yakutat collision in central Alaska, with local earthquakes and active source data: Journal of Geophysical Research, v. 111, B11303, doi:10.1029/2005JB004240.

Lucke, O.H., 2014, Moho structure of Central America based on three-dimensional lithospheric density modelling of satellite-derived gravity data: International Journal of Earth Science, v. 103, p. 1733-1745, DOI 10.1007/s00531-012-0787-y.

Yuan, X., Asch, G., Bataille, K., Bock, G., Bohm, M., Echtler, H., Kind, R., Oncken, O., and Wölbern, I., 2006, Deep seismic images of the Southern Andes, in Kay, S.M., and Ramos, V.A., eds., Evolution of an Andean margin: A tectonic and magmatic view from the Andes to the Neuquén Basin (35°–39°S lat): Geological Society of America Special Paper 407, p. 61–72, doi: 10.1130/2006.2407(03).

Zellmer, G.F., 2008, Some first-order observations on magma transfer from mantle wedge to upper crust at volcanic arcs: Geological Society of London Special Publication 304, p. 15–31.

Model Crust 1.0 is from Laske et al database available at the following site accessed August 2015 http://igppweb.ucsd.edu/~gabi/crust1.html.

Laske, G., Masters., G., Ma, Z. and Pasyanos, M., Update on CRUST1.0 - A 1-degree Global Model of Earth's Crust, Geophys. Res. Abstracts, 15, Abstract EGU2013-2658, 2013.

GEOCHEMISTRY

Geochemical Data for Pliocene to Quaternary rock analyses used to calculate Sr/Y. These analyses have been filtered to remove SiO2 wt. % < 55, SiO2 wt. % > 68, MgO wt. % < 1, MgO wt. % > 6, Rb/Sr > 0.2, Rb/Sr <0.05, and Sr/Y outliers using the Thomson Tau method. Data is from Geochemistry of Rocks of the Oceans and Continents database (GEOROC; http://georoc.mpch-mainz.gwdg.de/georoc/, accessed July 2015.
